# Airway pressure release ventilation as a recruitment maneuver in mechanically ventilated children with restrictive lung disease

**DOI:** 10.3389/fped.2025.1662233

**Published:** 2025-10-10

**Authors:** Mashael F. Alqahtani, Jessica E. Allen, Craig M. Johnson, Timothy M. Maul, Christine K. Koshel

**Affiliations:** ^1^ Division of Pediatric Critical Care Medicine, Nemours Children's Health, Orlando, FL, United States; ^2^ Department of Pediatric Critical Care Medicine, Kentucky Children's Hospital, University of Kentucky, Lexington, KY, United States; ^3^ Department of Radiology, Nemours Children's Health, Orlando, FL, United States; ^4^ Department of Cardiothoracic Surgery, Perfusionist at Nemours Children’s Health, Orlando, FL, United States

**Keywords:** pediatric critical care, neuromuscular disease (NMD), obesity, mechanical ventilation, restrictive lung disease, airway pressure release ventilation (APRV)

## Abstract

**Rationale:**

Restrictive lung disease is common in pediatric patients, leading to acute-on-chronic respiratory failure and the need for invasive mechanical ventilation. There is no consensus on lung recruitment maneuvers.

**Objectives:**

To examine the use of airway pressure release ventilation (APRV) in patients with restrictive lung disease as an effective open-lung tool maneuver.

**Methods:**

This single-center retrospective case series included patients with restrictive lung disease in a 28-bed pediatric intensive care unit from 2013 to 2024, who developed acute-on-chronic respiratory failure requiring invasive mechanical ventilation. Inclusion criteria were ventilation for at least 48 h, with at least 24 h in APRV. Descriptive statistics were performed.

**Measurements and main results:**

18 patient encounters met inclusion criteria. Subjects were divided into two groups: neuromuscular disease (14 encounters) and truncal obesity (4 encounters). Lung surface area improved significantly in the first 12–24 h of APRV use: neuromuscular group by 7,600 mm^2^ [95% CI, 4,000–11,000 mm^2^]; *p* < 0.001, and obesity group by 15,000 mm^2^ [95% CI, 3,000–27,000]; *p* = 0.018. Atelectasis improved at 12–24 h and 48 h from starting APRV, with mean differences of 14% [95% CI, 4%–24%]; *p* = 0.005 and 14% [95% CI, 3%–25%]; *p* = 0.009, respectively. As expected, oxygenation improved substantially in both groups.

**Conclusion:**

APRV is a safe and effective method for improving atelectasis and oxygenation in children with RLD related to neuromuscular conditions and obesity. Further high-quality prospective studies are needed to establish clear guidelines for its use.

## Introduction

Restrictive lung diseases (RLD) are a heterogeneous group of pulmonary disorders characterized by reduced distensibility of the lungs, impaired lung expansion, decreased lung volumes, and a diminished total lung capacity (1). Causes are intrinsic, including interstitial lung diseases, and extrinsic, due to limitations in chest wall movement, resulting in decreased thoracic compliance (1). In pediatrics, extrinsic causes predominate, and include genetic neuromuscular diseases (2), neuromuscular scoliosis associated with cerebral palsy (3–5), and obesity (6). As RLD can contribute to hypoventilation and pump failure of the diaphragm, when this form of chronic lung disease is exacerbated by concomitant acute respiratory infection, these patients are particularly prone to acute on chronic hypoxic and hypercarbic respiratory failure and need for invasive mechanical ventilation (2, 7, 8). Despite this knowledge and consensus regarding the need for respiratory care in this population, there is no guidance regarding what ventilator mode by which to invasively ventilate children with RLD.

Airway pressure release ventilation (APRV) was first described in 1987 as a mode of invasive pressure control mechanical ventilation utilizing an inverse inspiratory: exhalation time ratio, by which continuous positive airway pressure is maintained in the airways, with only short spontaneous ventilation releases throughout the respiratory cycle (9). This ventilator mode works physiologically by maintaining a higher mean airway pressure, recruiting available lung units with varying time constants, and allowing for the improvement of refractory hypoxemia (3, 4), APRV has become synonymous with the “open lung concept”, in which shear stress and atelectrauma are minimized in the mechanically ventilated patient by preventing the lung from fully de-recruiting (5, 10). These properties have helped to label APRV as a rescue mode of ventilatory support in moderate to severe pediatric acute respiratory distress syndrome (PARDS), in whom conventional modes of ventilation fail to improve hypoxemia, or in whom providers are concerned for ventilator-induced lung injury due to volutrauma or barotrauma (3, 6, 10). Despite knowledge regarding the physiology of APRV, and understanding the pulmonary mechanics of RLD, to our knowledge, use of APRV as a recruitment maneuver in mechanically ventilated children with RLD has not previously been described in the literature.

In our center, we have utilized APRV for patients with RLD due to neuromuscular conditions or truncal obesity receiving invasive mechanical ventilation. Anecdotally, we have seen an increase in both patient types in recent years. and observed an improvement in gas exchange and resolution of atelectasis within 24–48 h of transitioning to APRV. Therefore, we aim to test our hypothesis that APRV is beneficial as a recruitment strategy in the pediatric population with RLD. This is a retrospective study to evaluate the effect of APRV on ventilation and atelectasis in pediatric patients with RLD.

## Methods

This is a single-center retrospective case series in a 28-bed pediatric intensive care unit (PICU). Patients were identified via the EPIC electronic medical record (EMR) from those patients admitted to the Nemours Children's Hospital, Florida Pediatric Intensive Care Unit (PICU) in Orlando, FL during the time period between March 1, 2013 and March 1, 2024. Patients were included if they fulfilled the following inclusion criteria: (1) between the ages of 0–18 years old, (2) restrictive lung disease (scoliosis or kyphosis, obesity, cerebral palsy, or genetic neuromuscular disease: e.g., spinal muscular atrophy or Duchenne muscular dystrophy), (3) diagnosed with acute or acute on chronic respiratory failure, (4) required invasive positive pressure ventilation for a minimum of 48 h, at least 24 h of which on APRV. Patients were excluded if diagnosed with PARDS.

### APRV initiation protocol

APRV was delivered using the Siemens Maquet Servo-I or the Getinge Servo-U mechanical ventilators based on availability. For patients transitioning from conventional ventilation to APRV, two key parameters were obtained. First, the time constant was measured by performing an inspiratory hold followed by an expiratory hold; this value was calculated. The measured time constant was subsequently used to guide the setting of the release time (T_low_).

Initial parameters were then established as follows: P_High_ was set at the mean airway pressure (P_AW_) from conventional ventilation plus 5 cmH_2_O, serving as the primary determinant of oxygenation. T_High_ (inspiratory time) was selected based on patient age group: 3–6 s for adults, 3–5 s for pediatric patients, and 2–3 s for neonates. The duration of T_High_ determined the frequency of pressure releases, with more frequent releases facilitating carbon dioxide clearance.

**Figure 1:**
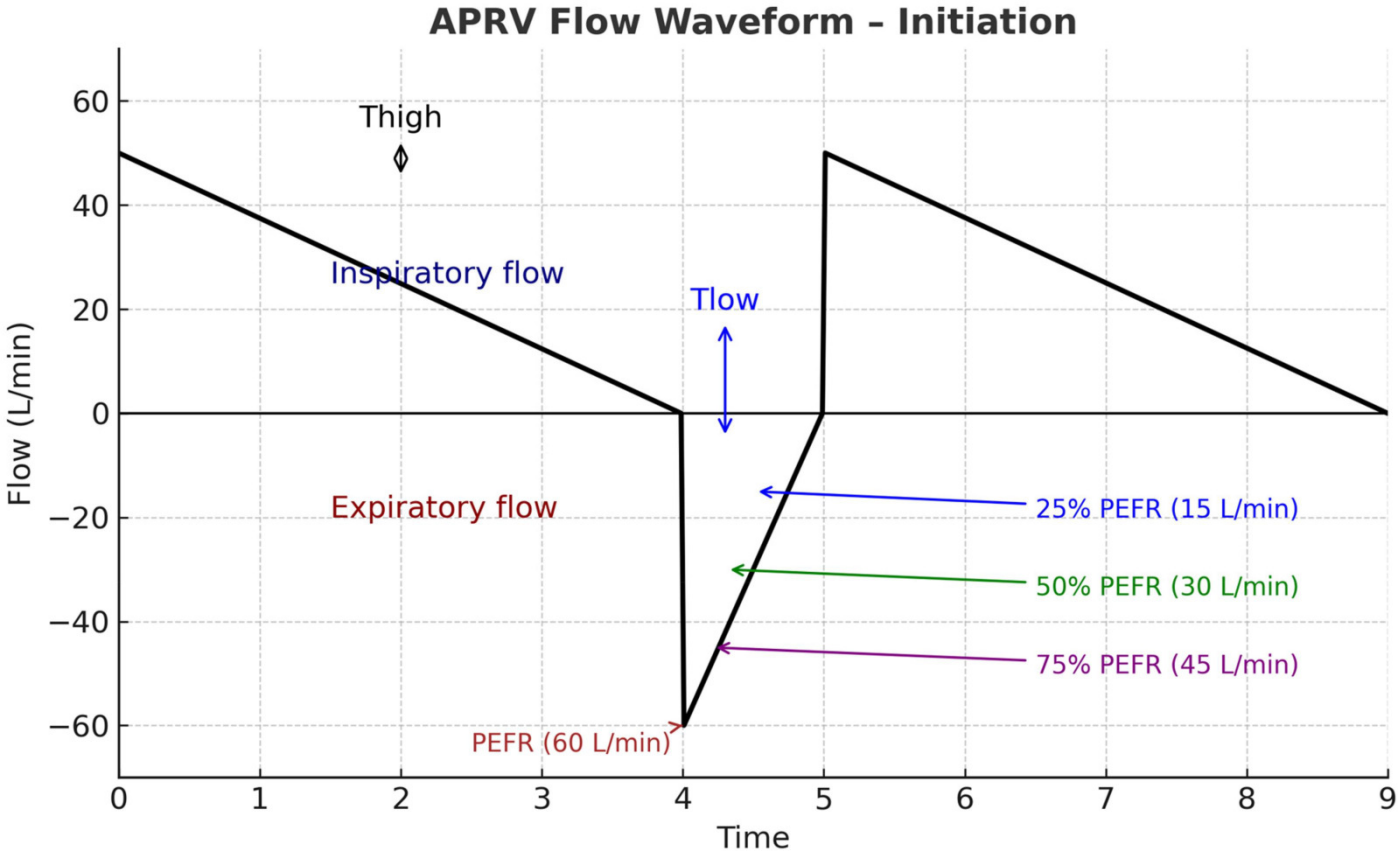
Illustration of APRV flow waveform initiation. The inspiratory phase (T_Hig**h**_) is characterized by a decelerating inspiratory flow above the baseline. During the release phase (T_Low)_ expiratory flow rapidly reaches the peak expiratory flow rate (PEFR, 60 L/min). Exhalation is intentionally terminated before full exhalation, commonly when expiratory flow declines to a set fraction of PEFR (ideally 50%), thereby maintaining intrinsic positive end-expiratory pressure and preventing alveolar derecruitment. APRV, airway pressure release ventilation; T_High,_ time at high pressure; T_Low_, time at low pressure; PEFR, peak expiratory flow rate.

The PEEP (P_Low)_ setting was always set to 0 cm H_2_O, with monitoring of total PEEP to assess intrinsic PEEP generation. Finally, the T_Low_ (release time at low pressure) was set equal to the patient's measured time constant, typically 0.2–0.8 s. Flow graphics were reviewed to confirm that exhalation terminated at 50%–75% of the peak expiratory flow rate, thereby ensuring adequate alveolar recruitment and minimizing de-recruitment Figure 1.

### Clinical/laboratory monitoring and data recording

**Figure 2:**
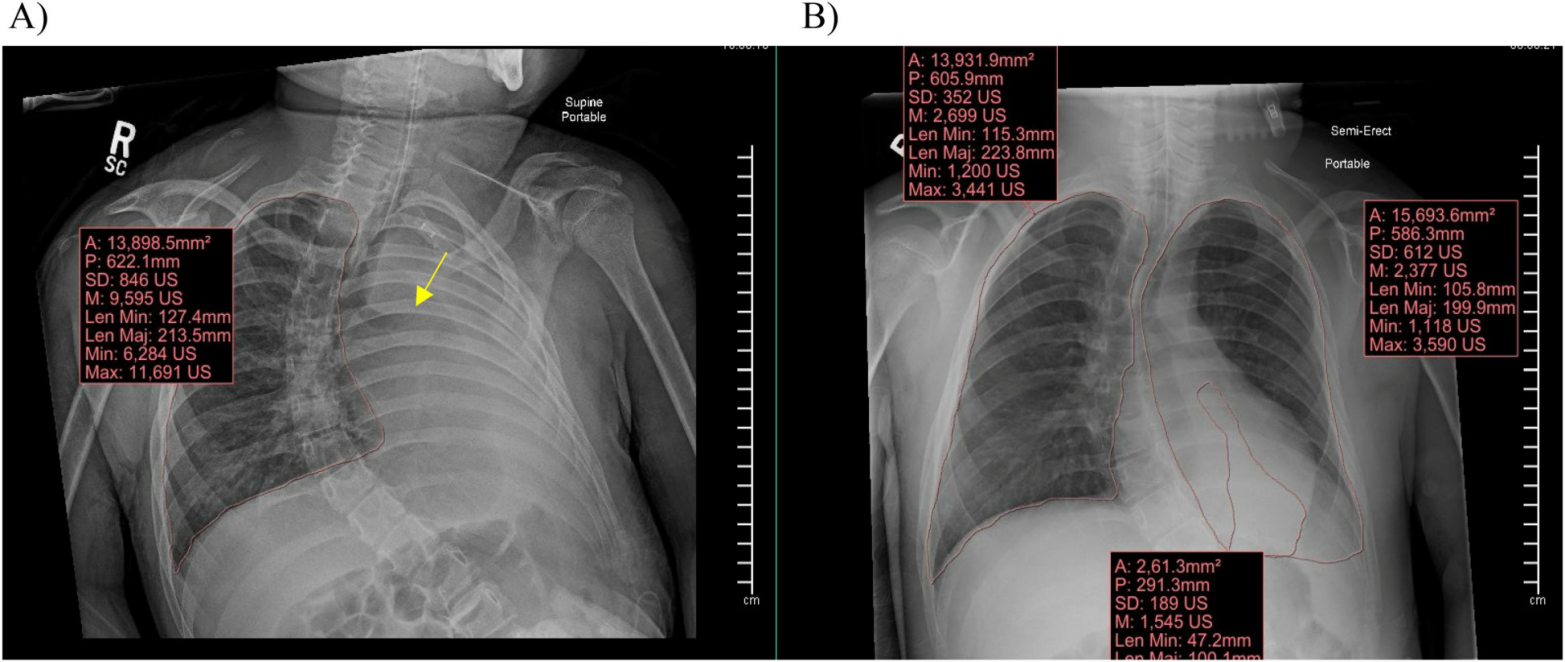
Synapse PACS system and the volume calculation tool used to calculate total lung surface area and area of atelectasis. **(A)** Demonstrates a clear fully expanded right lung with complete atelectasis of the left lung with abrupt cut off of the left mainstem bronchus (arrow). **(B)** Demonstrates a clear fully expanded right lung with partial atelectasis of the left lung with volumes demonstrated for quantification at each time point.

Information obtained included subjects’ demographic information (age, ethnicity, race, gender), height (cm), weight (kg), PICU length of stay (LOS) in days, cause of the RLD, and the admission diagnosis for which respiratory support was required. We collected chest x-rays for each patient upon admission to the PICU, prior to initiation of APRV (T0), and at times closest to 24 h (T1) and 48 h (T2) of APRV. The volume of atelectasis was calculated as a percentage of relative volumetric total lung volume by measuring the segmented atelectatic lung surface area (mm^2^) as compared to the total surface area of the expanded lung in each patient utilizing a region of interest (ROI) volume tool tracing with the AP portable chest radiographs at each time point for each encounter in the study. The volume of the fully expanded lung for each patient was calculated from the contralateral lung diaphragmatic excursion if there was not atelectasis or infiltrate and from prior chest radiographs in the case of bilateral involvement. All segmentation and calculations were performed by an experienced pediatric radiologist with 22 years of experience using Synapse PACS, FUJIFILM Inc. Lexington, MA, USA Figure 2. We documented ventilator settings including positive end-expiratory pressure (PEEP), inspired supplemental oxygen concentration (FiO2), and mean airway pressure (Paw) in children while on conventional ventilation, and maximum pressure delivered (Phigh), FiO2, and Paw in children receiving APRV. We recorded measurements of gas exchange, including partial pressures of oxygen and carbon dioxide (PaO2 and PaCO2) on arterial or venous blood gases, and utilized this information along with ventilator settings to calculate each child's Horowitz Index for Lung Function (P/F ratio) and oxygenation index (OI), to more accurately compare gas exchange while accounting for differences in patient size. We used oxygen saturation as measured by pulse oximetry (SpO2) as a substitute to calculate SpO2/F ratio and oxygenation saturation index (OSI) when arterial blood sampling was not available. Length of mechanical ventilation was collected, defined as the time from initiation of APRV to extubation in hours. We collected data regarding use of neuromuscular blockade, prone positioning, and receipt of bronchoscopy. We also described any documented complications while on APRV which could be attributed to this ventilation mode, primarily air leak syndrome or clinically significant hypotension requiring initiation of vasopressors.

### Statistical analysis

All statistical analysis was performed using SPSS (V28, IBM Corp, NY). Fixed data (demographics, pre-ventilator data, etc.) were maintained, while time-course data (ventilator settings, lung field analysis, and blood gas values at Time 0, 1, and 2) were rotated and analyzed longitudinally. All continuous variables were explored to determine normality. Patient weight, BMI, First P/F ratio, and PEEP on CMV prior to APRV were found to be not normally distributed by the Shapiro–Wilke test (*p* < 0.05) and compared using Mann–Whitney U testing and are presented as median and IQR. Normally distributed variables were analyzed by ANOVA and are presented as mean and standard deviation. Categorical variables are presented as *N* (%) and were compared using Fisher's Exact test. Longitudinal data were analyzed using generalized linear models with mixed effects and heterogenous autoregressive correlation to compare patient types. Statistical significance was set with alpha < 0.05.

## Results

A total of 18 patient encounters were identified that met inclusion criteria. In the neuromuscular group, there were 14 patient encounters, comprised of 11 unique patient encounters and 1 patient with 3 different encounters. In the obese group, there were 4 unique patient admission encounters. All patients were started on CMV (Control data) and were switched to APRV based on the clinical need for recruitment of atelectasis.

We divided the participants into two groups based on the causes of RLD physiology: the neuromuscular group and the obese group, as shown in Table 1. The obese group was older than the neuromuscular group, with an average age of 14 years (± 2.3) compared to 10.45 years (± 3.7); however, this difference was not statistically significant (*p* = 0.195). Demographic data indicated that height, weight, and body mass index (BMI) were significantly different between the two groups. The obese group was heavier and taller, with a weight of 127 kg (range: 94–169 kg) compared to 24.7 kg (range: 22.1–34.1 kg) in the neuromuscular group (*p* < 0.001). The average height for the obese group was 166.95 cm (±4.16 cm) vs. 112.72 cm (±15.2 cm) for the neuromuscular group (*p* < 0.001). The BMI for the obese group was 44.85 (range: 33.3–60.85) compared to 21.3 (range: 17.21–22.4) for the neuromuscular group (*p* = 0.003). Four patients in our neuromuscular group received continuous neuromuscular blockade. Three of the neuromuscular group were rotated to prone positioning during their intubation vs. one in the obese group. Bronchoscopy was performed to address mucous plugging and/or to obtain bronchoalveolar lavage samples in 7 patients in the neuromuscular group vs. one in the obese group (Table 1).

**Table 1:** Patient characteristics.

Variable	Neuromuscular (*n* = 14)	Obesity (*n* = 4)	*p*-value
Age, year	10.5 ± 3.7	14.0 ± 2.3	0.195
Weight, kg	24.7 (22.1–34.1)	126.8 (94.1–168.6)	<0.001
Height, cm	112.7 ± 15.2	167.0 ± 4.2	<0.001
BMI	21.3 (17.2–22.4)	44.9 (33.3–60.9)	0.003
Race			0.105
Other	11 (78.6)	1 (25.0)	
Black	1 (7.1)	1 (25.0)	
White	2 (14.3)	2 (50.0)	
Ethnicity			0.533
Hispanic/Latino	11 (78.6)	2 (50.0)	
Non-Hispanic	3 (21.4)	2 (50.0)	
Sex			1.0
Male	9 (64.3)	3 (75.0)	
Female	5 (35.7)	1 (25.0)	
Ventilator setting and gas exchange data Prior to APRV			
First pCO₂, mmHg	61.2 ± 19.1	55.5 ± 19.5	0.787
First PaO₂ or SaO₂	0.98 (0.95–0.98)	0.98 (0.96–1.0)	0.505
First P/F ratio (or SaO₂/F ratio)	98 (98–173)	144 (97.5–194)	0.733
First OI (or OSI)	6.8 ± 1.2	6.0 ± 1.1	0.537
PEEP on CMV prior to APRV, cmH₂O	9 (8–10)	9.5 (8–12.5)	0.798
FiO₂ on CMV prior to APRV	0.7 (0.45–1.0)	0.8 (0.55–1.0)	0.505
MAP on CMV prior to APRV, cmH₂O	14.6 ± 2.8	16.8 ± 7.7	0.487
Pre-pCO₂, mmHg	47.4 ± 11.6	49.5 ± 3.9	0.489
Pre-SaO₂ (if no PaO₂)	0.97 ± 0.02	1.0 ± 0.0	0.383
Pre-PaO₂, mmHg	93.4 ± 32.6	82.3 ± 16.5	0.522
Pre P/F ratio	152.0 ± 55.8	145.3 ± 62.6	0.624
Pre OSI	15.0 ± 5.5	6.0 ± 0.0	0.488
Pre OI	10.6 ± 5.7	14.8 ± 8.2	0.313
Respiratory support at admission			0.071
BiPAP	8 (57.1)	0 (0.0)	
MV	3 (21.4)	2 (50.0)	
HFNC	2 (14.3)	0 (0.0)	
Nasal cannula	1 (7.1)	1 (25.0)	
Oxygen mask	0 (0.0)	1 (25.0)	
Initial ventilation mode			0.533
PC	3 (21.4)	2 (50.0)	
PRVC	11 (78.6)	2 (50.0)	
Bronchoscopy	7 (50.0)	1 (25.0)	0.588
Neuromuscular blockade	4 (28.6)	0 (0.0)	0.524
Prone positioning	3 (21.4)	1 (25.0)	1.0
Complications	2 (14.3)	1 (25.0)	1.0

APRV, airway pressure release ventilation; P/F ratio, PaO_2_/FiO2 ratio; SaO_2,_ arterial oxygen saturation; OI, oxygenation index; OSI, oxygen saturation index; BiPAP, bilevel positive airway pressure; CMV, conventional mechanical ventilation; HFNC, high flow nasal cannula; PC, pressure control; PRVC, pressure regulated volume control.

Data are presented as median (interquartile range), mean ± SD, or *n* (%).

No differences were observed in other categorical variables between the two groups, including gender, race, initial blood gases, oxygenation, ventilation, and mode of ventilatory support. PICU LOS and LOS were not compared due to many confounding reasons. Our hospital does not have a step-down unit, and the majority of patients remain in the PICU due to shortage of acute care beds or due to their need of noninvasive ventilation or ventilation through tracheostomy, which prevents transfer. Individual patient-level data are provided in the Supplementary Materials (Supplementary Table 1).

### Outcomes

#### Ventilation outcomes

##### APRV resulted in functional recruitment by decreasing atelectasis and increasing lung surface area

**Figure 3:**
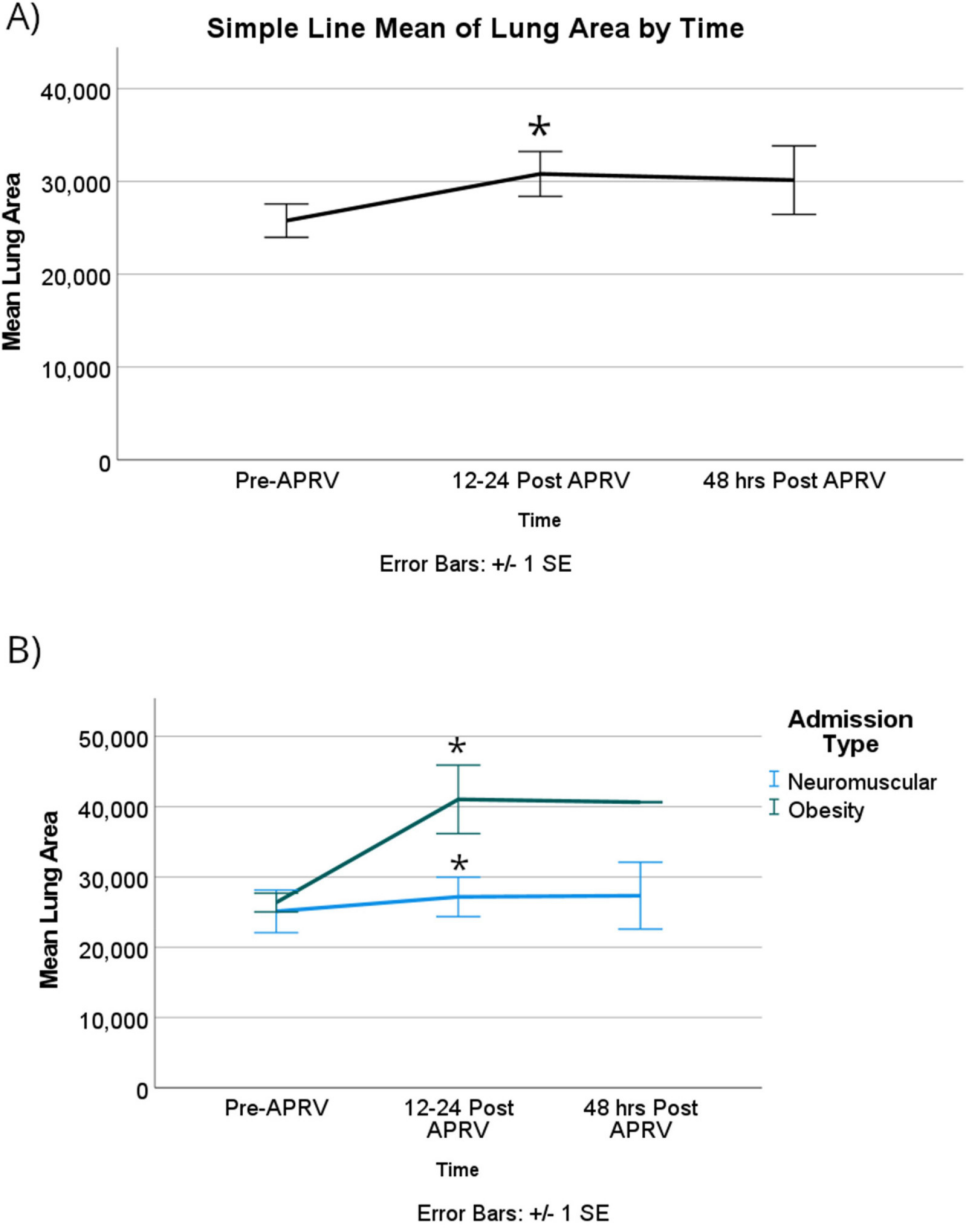
For lung surface area, **(A)** there was a significant improvement in the mean surface area among all patients with RLD at 12–24 h after starting APRV. **(B)** When patients were categorized based on their physiological cause of RLD, the obesity group showed a statistically significant improvement compared to the NMD group in the first 12–24 h. Denote pairwise *p* < 0.05.

**Figure 4:**
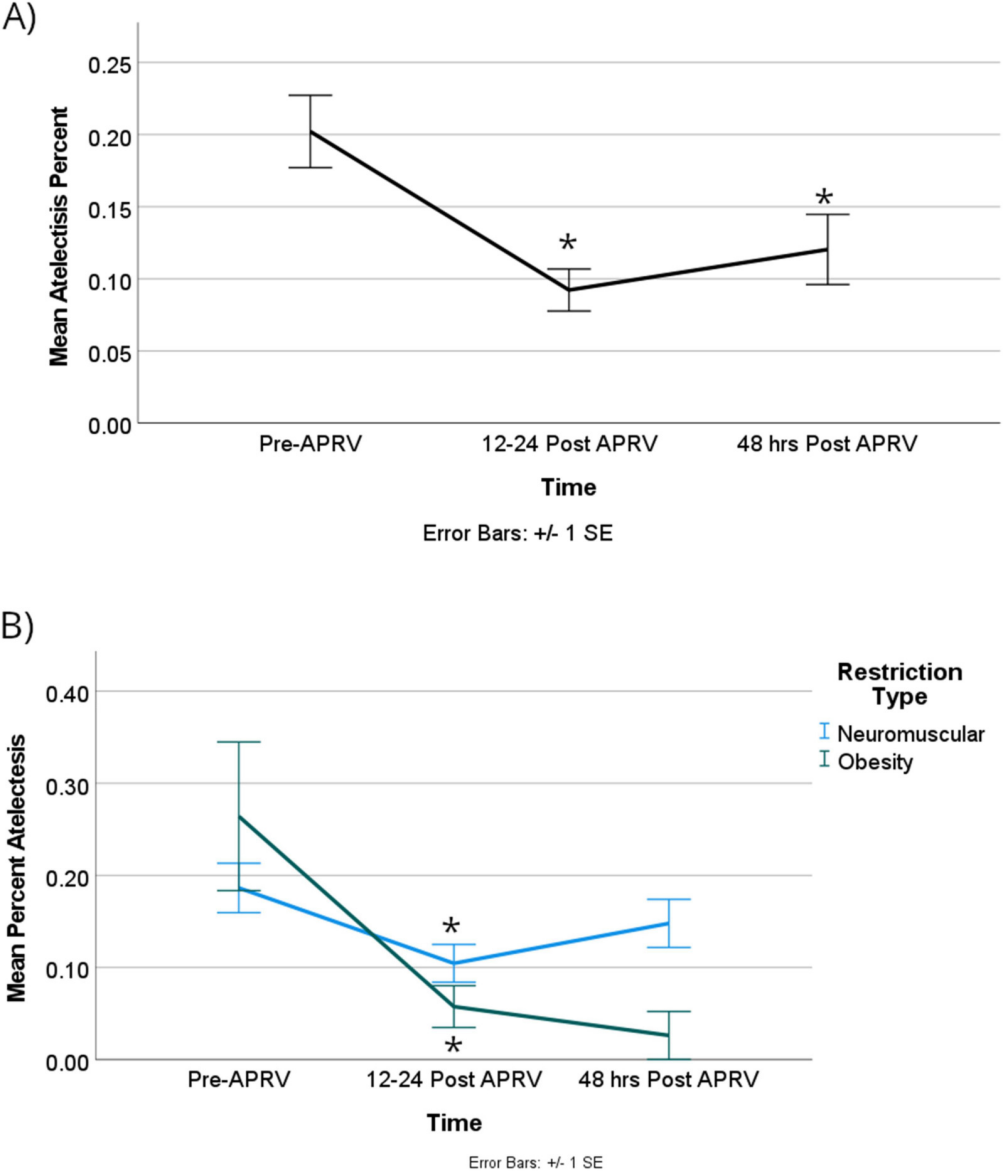
There was a significant difference in the percentage of atelectasis when the group was combined **(A)** between Pre-APRV and the 12–24 h, as well as pre-APRV and the 48 h post-APRV times, but not between 12 and 24 and 48 h post-APRV. **(B)** There was a significant difference between RDL physiologic categories at the first two time points (Pre-APRV and 12–24 h post-APRV). Denote pairwise *p* < 0.05.

For all patient encounters, there was a statistically significant improvement of atelectasis comparing T0 to T1, at a mean difference of about 14% [95% CI, 4%–24%]; *p* = 0.005. The lung surface area showed a statistically significant improvement comparing T0 to T1 of about 7,600 mm2, which equates to mean difference in atelectasis of 14% [95% CI, 4,000–11,000 mm^2^]; *p* < 0.001 (Figure 3A). There is also a statistically significant improvement of atelectasis from T0 to T2, again a mean difference of about 14% [95% CI, 3%–25%]; *p* = 0.009, respectively (Figure 4A). There was no significant improvement based on the restriction group difference (Figure 4B; Supplementary Index).

Again, the obese group showed a significantly improved lung surface area by 15,000 mm^2^ [95% CI, 3,000–27,000]; *p* *=* 0.018 (Figure 3B).

#### Oxygenation outcomes

##### APRV resulted in improvement in oxygenation over time in patients with restrictive lung disease without worsening ventilation

Oxygenation demonstrated statistical significance over time. The partial pressure of oxygen (PaO2) increased substantially and remained at an increased value for at least 48 h. Specifically, PaO2 increased by 63 mmHg [95% CI, 12.5–114]; *p* = 0.014 when comparing T0 with T1, and by 61 mmHg [95% CI, 11–111]; *p* = 0.017 from T1 to T2. Notably, there was no statistical significance difference between T0 and T2 (Supplementary Index).

**Figure 5:**
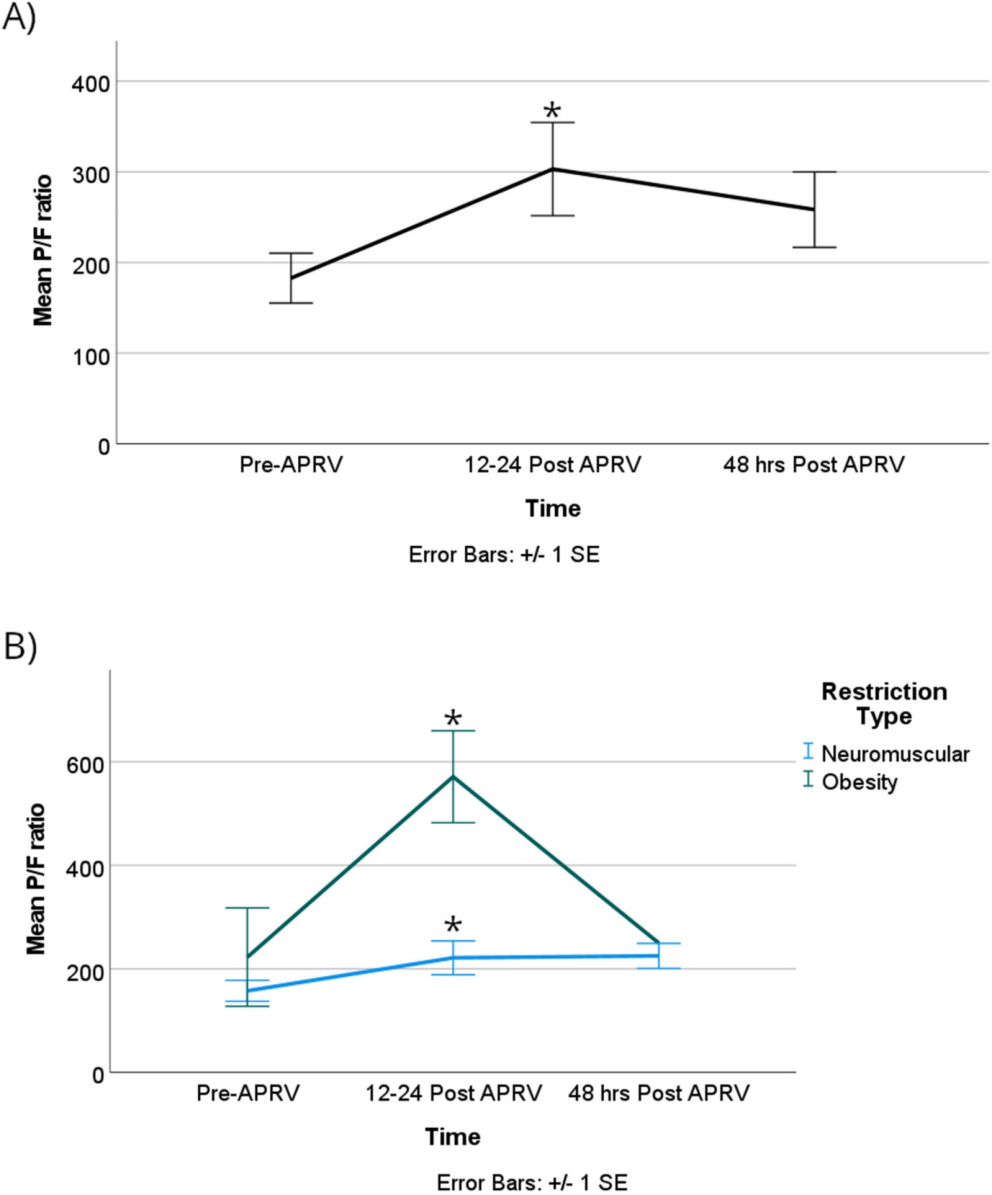
The P/F ratios were significantly improved in the first 12–24 h after starting APRV in the combined group **(A)** the same effect was seen when the group was divided based on RLD cause, with the obesity group showing a significant increase in P/F ratio compared to the NMD group during the first 12–24 h **(B)** denote pairwise *p* < 0.05.

The obese group demonstrated a statistically significant higher PaO2, with a mean difference of 121 mmHg [95% CI, 58–184]; *p* = 0.002. The PaO2/FiO2 ratio also showed statistically significant improvement, increasing by 193 mmHg from T0 to T1 [95% CI, 17–340]; *p* = 0.033 (Figure 5A). Although there was a change in the P/F from T1 to T2, it did not reach statistical significance with a *p*-value of 0.06 (Figure 5A; Supplementary Index). The obese group exhibited a statistically significant greater difference in the P/F ratio compared to the neuromuscular group, with a mean difference of 323 mmHg [95% CI, 142–504]; *p* = 0.005 (Figure 5B).

**Figure 6:**
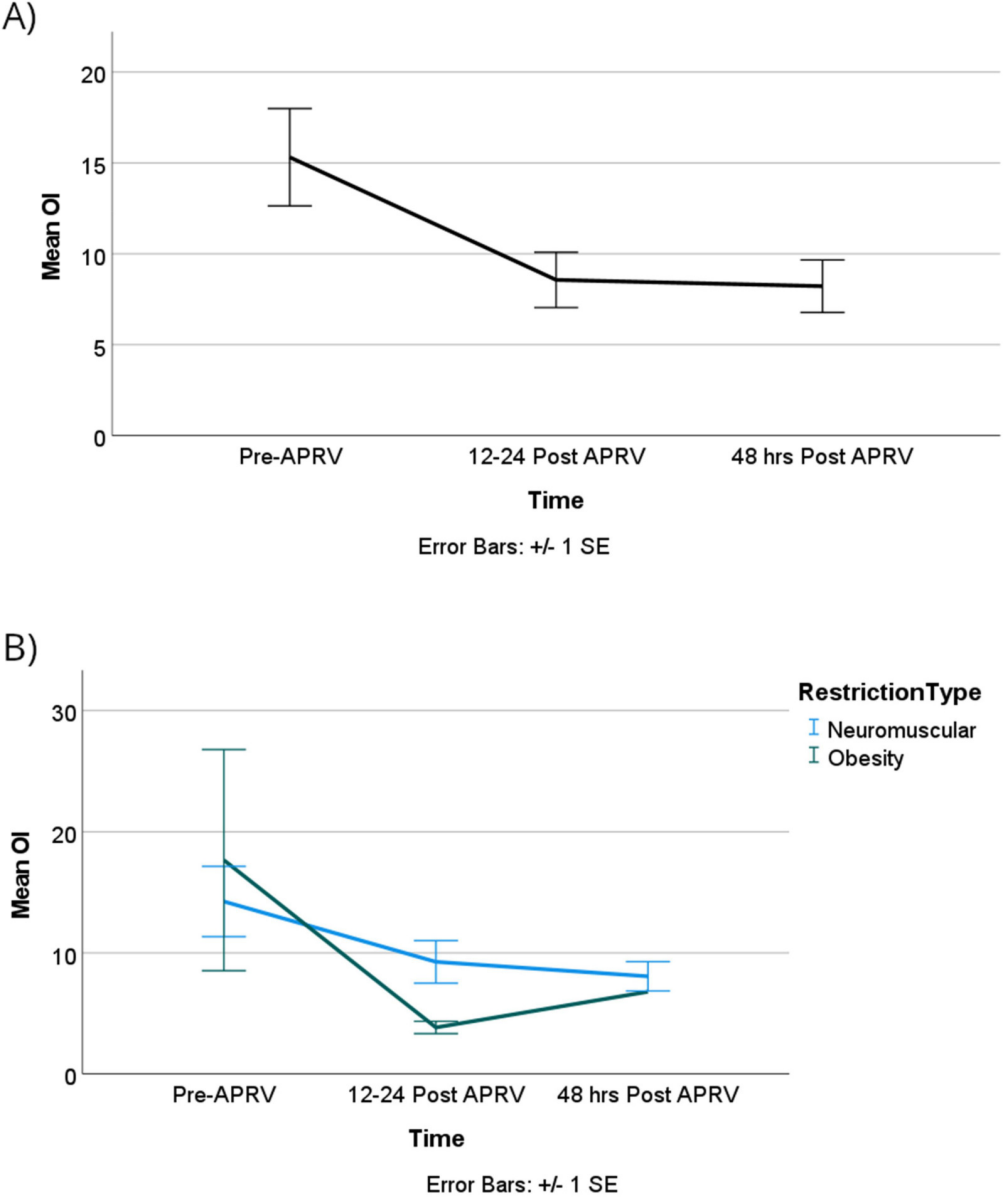
OI improved over time, but it did not reach statistical significance in all groups **(A)** or when categorized by RDL cause **(B)**.

**Figure 7:**
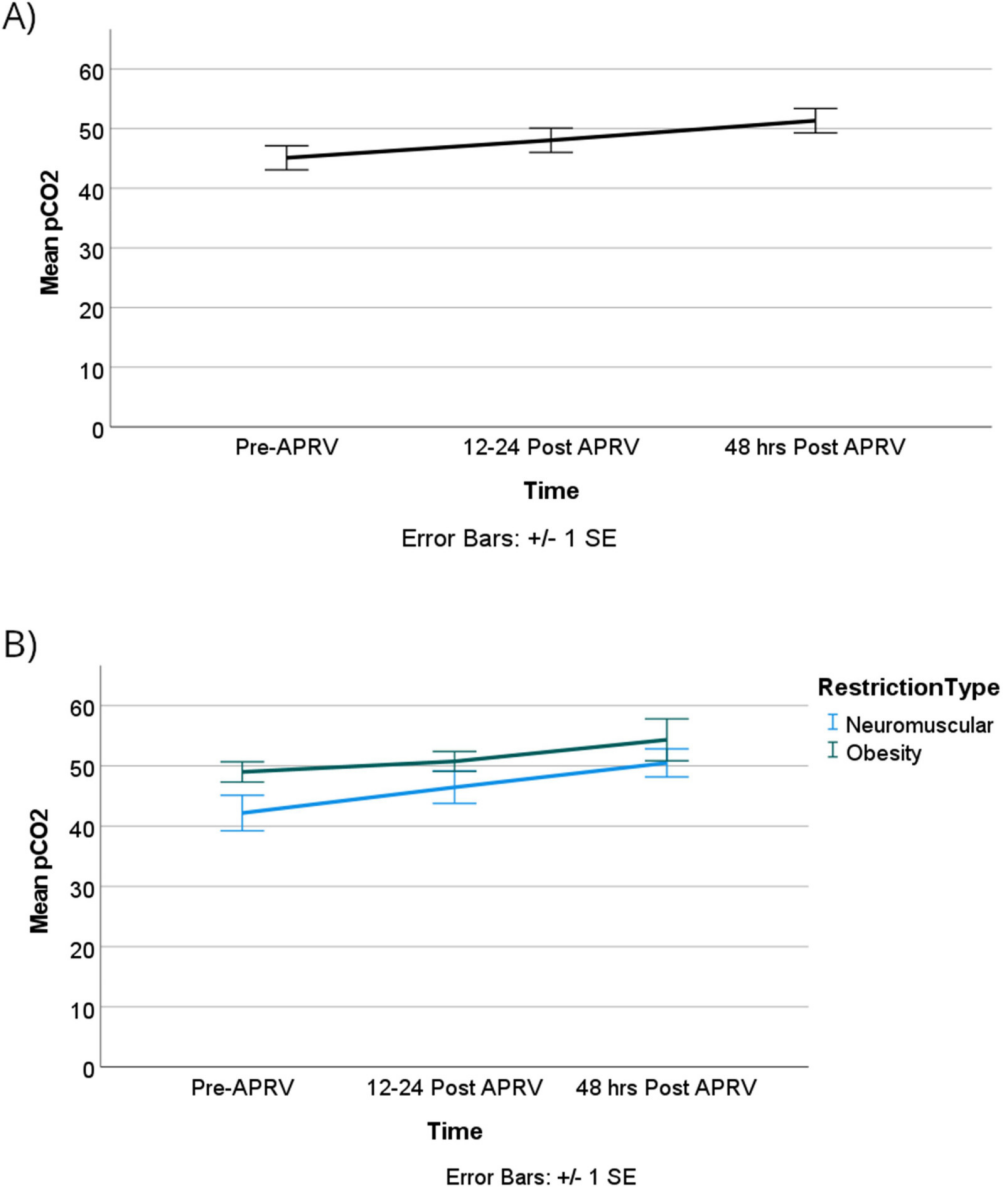
pCO_2_ was not impacted by time **(A)** or by RLD cause and time **(B)**.

The oxygenation index (OI) showed no statistically significant difference over time or between the two groups, however, as illustrated in Figure 6. OI improved from T0 to T1 with more clinically significant improvements observed in the obese group compared to the neuromuscular group, although these differences were not statistically significant. Lastly, our patients did not show worsening of ventilation over time or based on the physiology of the restrictive lung disease as shown in Figure 7.

## Discussion

Our case series of 18 patient encounters, 15 unique patients, showed that patients with RLD and with acute respiratory failure, without the diagnosis of PARDS, who were transitioned from conventional mechanical ventilation (CV) to APRV showed an increase in lung volume, decrease in atelectasis, and improvement in oxygenation within the first 24 h of transition. APRV is a ventilator mode that when employed in pediatrics, is most often utilized as a rescue modality to improve gas exchange in PARDS. Our study showed that for children with RLD and atelectasis impairing gas exchange and/or their ability to liberate from mechanical ventilation, APRV can be successfully utilized as a recruitment modality. Use of APRV in this patient population reduces atelectasis and thereby increases the amount of lung surface area able to participate in gas exchange. This results in improved oxygenation for up to 24 h after the introduction of APRV without a clinically significant worsening of existing ventilation impairment. Our study also demonstrates that ARPV is safe and well-tolerated, even in patients receiving neuromuscular blockade.

RLD in the pediatric age group is most often encountered in children with neuromuscular disease, either due to cerebral palsy or muscular dystrophies. It is often in association with neuromuscular scoliosis, which imposes additional restrictions. With the increase in childhood obesity, critical care providers are seeing more adolescents with morbid obesity, with RLD resulting from their chest wall and abdominal adiposity. Despite this increasingly common group of patients, children with neuromuscular disease still comprised the majority of our cohort.

Alveolar recruitment maneuvers are performed by respiratory therapists to reduce atelectasis and improve gas exchange in children with acute lung injury. Methods in the literature by which to transiently provide increased transpulmonary pressure for recruitment include sustained high-pressure inflation techniques, intermittent sigh breaths, and brief periods of increased PEEP (11). Criticisms of these recruitment maneuvers include the risk of barotrauma, air leak, and hemodynamic instability, therefore prone positioning has emerged as an attractive alternative. There is currently no consensus regarding the use of recruitment maneuvers in pediatrics, with the literature showing conflicting results and inconsistent survival benefits (12). Our study showed that patients with atelectasis due to RLD experienced on average a 14% reduction in atelectasis after 12–24 h of APRV, which they maintained after 48 h of APRV despite weaning the mean airway pressure. This suggests that recruitment is sustained with APRV, even after decreases in ventilatory support.

In terms of oxygenation, both PaO2 and the P/F ratio were significantly improved at T1, findings that were most significant in children whose RLD was attributable to obesity. While the OI also improved at T1, as expected, the improvement was just short of achieving statistical significance. A possible explanation for this finding is that in efforts to re-expand atelectatic lung, we may have provided more mean airway pressure than was needed, influencing the OI equation. We postulate that concomitant obstructive lung disease from mucous plugging and microatelectasis may explain why children with neuromuscular disease did not have the same improvement in oxygenation as obese children in whom this modality was used. Use of APRV did not significantly worsen ventilation impairment, which is important to note since this modality is sometimes criticized for its inefficient ventilation.

Complications occurred rarely in our cohort. One obese child developed pneumomediastinum, which was not clinically significant, and one neuromuscular child developed worsening hypercapnic respiratory failure, necessitating a change in ventilatory mode. A second neuromuscular child developed an air leak prior to being placed in APRV, which, importantly, did not preclude the decision to utilize this modality.

### Limitation

There are a few study limitations that must be discussed. First, this is a retrospective study of previous patient encounters over multiple years. As such, we could not control the ventilator settings that were utilized and if or when blood gases or chest radiographs were performed, limiting standardization of patient comparison. The decision to utilize APRV for recruitment was at the discretion of the critical care provider, and since our unit does not have a protocol for initial APRV settings or how to wean support, these practices were also at the discretion of the critical care provider and respiratory therapist. Concomitant recruitment modalities and airway clearance therapies were common in our cohort, which may have confounded our results. Many of our patients were also placed in the prone position and several of the neuromuscular children had bronchoscopies for diagnostic and/or therapeutic benefit. Notably, some were placed in APRV for alveolar recruitment after the development of atelectasis that followed bronchoscopy. Lastly, this study was underpowered, particularly in the obese group, which limits our ability to provide concrete guidelines for patient care.

## Conclusion

APRV is a safe, effective way to provide alveolar recruitment and lung volume optimization in children with RLD from both neuromuscular conditions and obesity, and thus, improves oxygenation. Our results suggest APRV should be considered for lung recruitment in patients with RLD and recurrent atelectasis delaying extubation attempts. In order to provide recommendations and clear guidelines as to its use in this population, however, additional high-powered prospective studies utilizing an APRV protocol are necessary. To overcome our limitations, we propose conducting a prospective case control feasibility study to evaluate the benefits of APRV over conventional mechanical ventilation in the pediatric RLD population in increasing ventilator-free days and shortening the PICU LOS.

## Data Availability

The original contributions presented in the study are included in the article/Supplementary Material, further inquiries can be directed to the corresponding author.
