# Supplementary material for: Airway pressure release ventilation as a recruitment maneuver in mechanically ventilated children with restrictive lung disease
Source: Front Pediatr. 2025 Oct 10;13:1662233. doi: 10.3389/fped.2025.1662233 (PMC12549264; doi:10.3389/fped.2025.1662233)
Supplement: Supplementary file 1 [file Table1.docx]

| \| **Patient** \| **Age (y), Gender** \| **Weight, Race/Ethnicity** \| **Type of RLD** \| **PICU admission dx** \| **VENT settings Closest to APRV introduction** \| **Pre OI & PF** \| **APRV settings** \| **Post OI & PF** \| **Sedation Infusions (Opioid/Benzo/Alpha-2 agonist /Propofol),**  **NMB** \| **Bronchoscopy or**  **prone** \| **Complications** \| \| --- \| --- \| --- \| --- \| --- \| --- \| --- \| --- \| --- \| --- \| --- \| --- \| \| 1a \| 14, F \| 23.6kg,  Other race/  Another Hisp \| NM \| Bacterial Pneumonia \| PC  PEEP:10  FIO2:0.7  MAP:17  i-time:1  IMV:20  PC:20 \| S:F 87  OI: 19.5 \| P:26/0  T:5/0.4  MAP:23  Fio2:0.6 \| P:F100  OI: 23 \| Alpha 2 agonist  Propofol  Benzo (intermittent)  NMB \| Bronch \| None \| \| 1b \| 14, F \| 23.6kg,  Other race/  Another Hisp \| NM \| Bacterial Pneumonia \| PRVC  PEEP:9  FIO2:0.7  MAP:15  i-time:0.9  IMV:16  TV:160 \| P:F 107  OI: 14 \| P:26/3  T:4/0.4  MAP:25  Fio2:0.7 \| P:F 164  OI:9 \| Opioid  Alpha 2 agonist  Benzo (intermittent) \| Bronch \| None \| \| 2 \| 13, F \| 44.5kg,  Other race/  Another Hisp \| NM \| Bacterial Pneumonia \| PRVC  PEEP:9  FiO2:0.55  MAP:12  i-time:1  IMV:18  TV:270 \| P:F127  OI:9.4 \| P:22/0  T:4.5/0.5  MAP:20  FiO2:0.6 \| P:F138  OI: 14 \| Opioid  Alpha 2 agonist  Benzo (intermittent) \| Bronch  Prone \| Inability to ventilate (PCO2 110) \| \| 3 \| 15, M \| 101.9 kg,  White/  Non-Hisp \| Obesity \| Acute GI bleed \| PRVC  PEEP:10  FiO2:0.6  MAP:11  i-time:1  IMV:12  TV:500 \| P:F170  OI:6.5 \| P:24/0  T: 3/0.3  MAP:21  FiO2:0.6 \| P:F207  OI:10.2 \| Opioid  Alpha 2 agonist \| None \| Pneumomediastinum \| \| 4 \| 7, M \| 22.1 kg,  White/  Non-Hisp \| NM \| Viral Pneumonia \| PRVC  PEEP:8  FiO2:0.6  MAP:12  i-time:0.8  IMV:16  TV:150 \| P:F112  OI:10.7 \| P:20/1  T:4/0.6  MAP:11  FiO2:0.6 \| P:F155  OI:7.1 \| Opioid  Alpha 2 agonist  Benzo (intermittent)  NMB \| Prone \| None \| \| 6a \| 7, M \| 25.3 kg,  Other race/  Puerto Rican \| NM \| Viral Pneumonia \| PC  PEEP:8  FiO2:1  MAP:15  i-time:1  IMV:20  PC:22 \| S:F:173  OI:6.03 \| P:24/0  T:3/0.3  MAP:22  FiO2:1 \| P:F94  OI:23.4 \| Propofol \| Bronch (pre-APRV) \| None \| \| 6b \| 7, M \| 23.7 kg,  Other race/  Puerto Rican \| NM \| Viral Pneumonia \| PRVC  PEEP:10  FiO2:0.7  MAP:14  i-time:0.9  IMV:20  TV:140 \| S:F141  OSI:10 \| P:21/1  T:4/0.5  MAP:19  FiO2:0.65 \| S:F 147  OSI:12.9 \| Opioid \| None \| None \| \| 6c \| 9, M \| 25.4 kg,  Other race/  Puerto Rican \| NM \| Bacterial Pneumonia  Sepsis  Septic shock \| PRVC  PEEP:8  FiO2:0.4  MAP:12  i-time:1  IMV:15  TV:150 \| P:F 208  OI:5.8 \| P:24/0  T:3/0.4  MAP:24  FiO2: \| P:F 250  OI: 9.6 \| Alpha 2 agonist \| Bronch (pre-APRV) \| None \| \| 6d \| 9, M \| 24.7 kg,  Other race/  Puerto Rican \| NM \| Viral Pneumonia \| PRVC  PEEP:10  FiO2:1  MAP:19  i-time:0.9  IMV:26  TV:180 \| S:F 95  OSI:20 \| P:24/0  T:5/0.7  MAP:20  FiO2:0.8 \| P:F 191  OI:10.5 \| Opioid \| None \| None \| \| 7 \| 14, M \| 34.1 kg,  Other race/  Another Hisp \| NM \| Bacterial Pneumonia \| PRVC  PEEP:10  FiO2:0.5  MAP:13  i-time:1.3  IMV:12  TV:250 \| P:F 202  OI:6.4 \| P:22/0  T:4/0.5  MAP:20  FiO2:0.6 \| P:F 168  OI:11.9 \| Opioid  Alpha 2 agonist  Benzo (intermittent) \| Bronch \| None \| \| 8 \| 17, M \| 86.2 kg,  Other race/  Another Hisp \| Obesity \| Bacterial sepsis \| PC  PEEP:7  FiO2:0.5  MAP:11  i-time:1.1  IMV:18  PC:12 \| S:F 200  OSI:6 \| P:20/0  T:4.5/0.5  MAP:19  FiO2:0.5 \| S:F 198  OSI: 9.6 \| Opioid  Alpha 2 agonist  Anesthetic  Benzo (intermittent +infusion) \| None \| None \| \| 10 \| 13, F \| 185.5 kg,  Black/  Non-Hisp \| Obesity \| T&A \| PRVC  PEEP:9  FiO2:1  MAP:16  i-time:1.2  IMV:16  TV:500 \| P:F 64  OI:25 \| P:25/1  T:6/0.65  MAP:24  FiO2:1 \| P:F 67  OI:35.8 \| Anesthetic  Alpha 2 agonist \| Prone \| None \| \| 11 \| 15, F \| 37.7 kg,  White/  Another Hisp \| NM \| Covid-19 Pneumonia \| PC  PEEP:10  FiO2:0.6  MAP:18  i-time:1  IMV:20  PC:26 \| P:F 140  OI: 12.9 \| P: 28/0  T:3/0.3  MAP:27  FiO2:0.7 \| P:F 109  OI: 24.9 \| Opioid  Alpha 2 agonist \| None \| None \| \| 13 \| 15, M \| 41.6 kg,  Other race/  Another Hisp \| NM \| Covid-19 Pneumonia \| PC  PEEP: 10  FiO2:0.5  MAP:18  i-time:1  IMV:24  PC:24 \| P:F 356  OI: 5.1 \| P: 24/0  T: 4/0.4  MAP: 23  FiO2: 0.5 \| P:F 356  OI: 6.5 \| Opioid  Alpha 2 agonist  NMB \| Bronch \| None \| \| 14 \| 11, M \| 151.7 kg,  White/  Cuban \| Obesity \| T&A \| PRVC  PEEP: 15  FiO2:1  MAP:29  i-time:1.2  IMV:10  TV: 600 \| P:F 202  OI: 12.8 \| P: 28/0  T:3.5/0.3  MAP:26  FiO2:0.4 \| P:F 395  OI:7 \| Anesthetic  Alpha 2 agonist \| Bronch \| None \| \| 15 \| 7, F \| 13.1 kg,  Black/  Non-Hisp \| NM \| Bowel obstruction \| PRVC  PEEP: 8  FiO2: 0.4  MAP:17  i-time:0.8  IMV:30  TV:110 \| S:F 245  OSI:7 \| P: 25/1  T: 4/0.5  MAP: 22  FiO2: 0.5 \| S:F 248  OI:11 \| None \| Bronch \| None \| \| 16 \| 15, M \| 64.5 kg,  Other race/  Another Hisp \| NM \| Aspiration pneumonia \| PRVC  PEEP: 12  FiO2:1  MAP:18  i-time:1  IMV:18  TV:450 \| P:F 68  OI: 26.5 \| P: 32/0  T:3/0.55  MAP:27  FiO2:1 \| P:F 75  OI: 36 \| Opioid  Alpha 2 agonist  NMB \| Prone \| None \| \| 17 \| 9, F \| 20.6 kg,  Other race/  Another Hisp \| NM \| Sepsis, Septic Shock \| PRVC  PEEP:6  FiO2:0.45  MAP:10  i-time:0.9  IMV:8  TV:140 \| P:F 262  OI: 3.8 \| P: 15/0  T:3.5/0.2  MAP:15  FiO2:0.5 \| P:F 186  OI:8.1 \| Alpha 2 agonist \| None \| None \| \| 18 \| 17, M \| 50.7 kg,  Other race/  Non Hisp \| NM \| Covid-19  Pneumonia \| PRVC  PEEP:9  FiO2:1  MAP:14  i-time:1.2  IMV:14  TV:300 \| P:F 156  OI: 9 \| P: 26/0  T:4/0.3  MAP:25  FiO2:1 \| P:F 225  OI:11.1 \| Opioid  NMDA receptor agonist  Benzo (intermittent)  Alpha 2 agonist \| None \| Airleak prior to APRV \| |
| --- | --- | --- | --- | --- | --- | --- | --- | --- | --- | --- | --- | --- | --- | --- | --- | --- | --- | --- | --- | --- | --- | --- | --- | --- | --- | --- | --- | --- | --- | --- | --- | --- | --- | --- | --- | --- | --- | --- | --- | --- | --- | --- | --- | --- | --- | --- | --- | --- | --- | --- | --- | --- | --- | --- | --- | --- | --- | --- | --- | --- | --- | --- | --- | --- | --- | --- | --- | --- | --- | --- | --- | --- | --- | --- | --- | --- | --- | --- | --- | --- | --- | --- | --- | --- | --- | --- | --- | --- | --- | --- | --- | --- | --- | --- | --- | --- | --- | --- | --- | --- | --- | --- | --- | --- | --- | --- | --- | --- | --- | --- | --- | --- | --- | --- | --- | --- | --- | --- | --- | --- | --- | --- | --- | --- | --- | --- | --- | --- | --- | --- | --- | --- | --- | --- | --- | --- | --- | --- | --- | --- | --- | --- | --- | --- | --- | --- | --- | --- | --- | --- | --- | --- | --- | --- | --- | --- | --- | --- | --- | --- | --- | --- | --- | --- | --- | --- | --- | --- | --- | --- | --- | --- | --- | --- | --- | --- | --- | --- | --- | --- | --- | --- | --- | --- | --- | --- | --- | --- | --- | --- | --- | --- | --- | --- | --- | --- | --- | --- | --- | --- | --- | --- | --- | --- | --- | --- | --- | --- | --- | --- | --- | --- | --- | --- | --- | --- | --- | --- | --- | --- | --- | --- | --- | --- | --- | --- | --- | --- | --- | --- | --- | --- | --- | --- | --- | --- | --- | --- | --- | --- |
