# Supplementary material for: Airway pressure release ventilation as a recruitment maneuver in mechanically ventilated children with restrictive lung disease
Source: Front Pediatr. 2025 Oct 10;13:1662233. doi: 10.3389/fped.2025.1662233 (PMC12549264; doi:10.3389/fped.2025.1662233)

APRV Study

# Description

This is an analysis of the APRV data provided by the PICU team. The goal was to analyze the resultant paO2, PF ratios, and OI when going from standard ventilator modes to 24 hr and 48hrs on APRV. There were two types of patients – infectious and non-infectious – that were coded by the PICU team.

# Statistical Analysis

All statistical analysis was performed using SPSS (V28, IBM Corp, NY). The data was read into SPSS from the Excel file. Fixed data (demographics, pre-ventilator data, etc) were maintained, while time-course data (Time 0, 1, 2) were rotated and analyzed longitudinally. All data was explored to determine normality. Patient weight, BMI, First PaO2, First P/F ratio, PEEP on CMV Prior to APRV, and FiO2 on CMV prior to APRV were found to be not normally distributed by the Kolmogorov-Smirnov Test (p<0.05). These are presented as median and IQR while the remainder are presented as mean and standard deviation. Categorical variables are presented as N (%). There 14 patient encounters (11 unique patients) included in the Neuromuscular group and 4 in the obesity group. Weight, height, and BMI were statistically different between the two groups.

|  |  | Neuromuscular (N=14) | Obesity (N=4) | p-value |
| --- | --- | --- | --- | --- |
|  | Age (yr) | 10.45 +3.66 | 14 +2.34 | 0.195 |
|  | **Weight (kg)** | **24.7 (22.1-34.1)** | **126.8 (94.05-168.6)** | **<0.001** |
|  | **Height (cm)** | **112.72 +15.2** | **166.95 +4.16** | **<0.001** |
|  | **BMI** | **21.3 (17.21-22.4)** | **44.85 (33.3-60.85)** | **0.003** |
|  | First pCO2 | 61.18 +19.08 | 55.5 +19.51 | 0.787 |
|  | First PaO2 or SaO2 | 0.98 (0.95-0.98) | 0.98 (0.96-1) | 0.505 |
|  | First P/F ratio (or SaO2/F ratio) | 98 (98-173) | 144 (97.5-194) | 0.733 |
|  | First OI (or OSI) | 6.84 +1.17 | 6 +1.1 | 0.537 |
|  | PEEP on CMV prior to APRV | 9 (8-10) | 9.5 (8-12.5) | 0.798 |
|  | FiO2 on CMV prior to APRV | 0.7 (0.45-1) | 0.8 (0.55-1) | 0.505 |
|  | MAP on CMV prior to APRV | 14.64 +2.75 | 16.75 +7.69 | 0.487 |
|  | pre PCO2 | 47.4 +11.63 | 49.5 +3.94 | 0.489 |
|  | pre-SaO2 (if no PaO2) | 0.97 +0.02 | 1 +0 | 0.383 |
|  | pre PaO2 | 93.43 +32.59 | 82.33 +16.48 | 0.522 |
|  | pre saO2/F ratio | 118 +25.2 | 200 +0 | 0.288 |
|  | pre P/F | 152.04 +55.84 | 145.33 +62.55 | 0.624 |
|  | pre-OSI | 15 +5.48 | 6 +0 | 0.488 |
|  | pre OI | 10.62 +5.72 | 14.76 +8.16 | 0.313 |
| Race | Other Race | 11 (78.6) | 1 (25) | 0.105 |
|  | Black | 1 (7.1) | 1 (25) |  |
|  | White | 2 (14.3) | 2 (50) |  |
| Ethnicity_Binary | Hispanic, Latino, or Spanish Origin | 11 (78.6) | 2 (50) | 0.533 |
|  | Non-Hispanic | 3 (21.4) | 2 (50) |  |
| Sex | M | 9 (64.3) | 3 (75) | 1 |
| Resp support at admission | bipap | 8 (57.1) | 0 (0) | 0.071 |
|  | CMV | 3 (21.4) | 2 (50) |  |
|  | HFNC | 2 (14.3) | 0 (0) |  |
|  | NC | 1 (7.1) | 1 (25) |  |
|  | oxy mask | 0 (0) | 1 (25) |  |
| Initial vent mode | PC | 3 (21.4) | 2 (50) | 0.533 |
|  | PRVC | 11 (78.6) | 2 (50) |  |
|  | Bronch (Y/N) | 7 (50) | 1 (25) | 0.588 |
|  | NMB (Y/N) | 4 (28.6) | 0 (0) | 0.524 |
|  | Proned simultaneously (Y/N) | 3 (21.4) | 1 (25) | 1 |
|  | Complications | 2 (14.3) | 1 (25) | 1 |

# Linear Mixed Modeling

Since the data was longitudinal and the variables of interest (paO2, OI and P/F Ratio) were normally distributed, a generalized linear model with mixed effects and heterogeneous autoregressive correlation was used to compare patient type (infectious vs non-infectious patients), general time based differences, and the interaction between patient type and time.

## paO2 model

The paO2 model found significant differences for Time, and the interaction between patient group and time.

| **Type III Tests of Fixed Effects^a^** | | | | |
| --- | --- | --- | --- | --- |
| Source | Numerator df | Denominator df | F | Sig. |
| Intercept | 1 | 12.235 | 154.335 | <.001 |
| **Time** | **2** | **13.713** | **9.001** | **.003** |
| Restrictive_binary | 1 | 12.235 | 4.348 | .059 |
| **Restrictive_binary * Time** | **2** | **13.713** | **7.857** | **.005** |
| a. Dependent Variable: Time 0 PaO2. | | | | |

Pairwise comparisons between time points indicate that Time 1 (12-24 hours after APRV) was significantly different from the baseline time as well as 48 hours post APRV, but 48 hours was not different from baseline.

| **Pairwise Comparisons^a^** | | | | | | | | |
| --- | --- | --- | --- | --- | --- | --- | --- | --- |
| (I) Time | (J) Time | Mean Difference (I-J) | Std. Error | df | Sig.^c^ | 95% Confidence Interval for Difference^c^ | |  |
|  |  |  |  |  |  | Lower Bound | Upper Bound |  |
| **Pre-APRV** | **12-24 Post APRV** | **-63.268^*^** | **18.564** | **13.297** | **.014** | **-114.080** | **-12.456** |  |
|  | 48 hrs Post APRV | -2.235 | 22.027 | 16.299 | 1.000 | -60.990 | 56.519 |  |
| **12-24 Post APRV** | **Pre-APRV** | **63.268^*^** | **18.564** | **13.297** | **.014** | **12.456** | **114.080** |  |
|  | **48 hrs Post APRV** | **61.032^*^** | **18.085** | **11.859** | **.017** | **10.671** | **111.394** |  |
| 48 hrs Post APRV | Pre-APRV | 2.235 | 22.027 | 16.299 | 1.000 | -56.519 | 60.990 |  |
|  | **12-24 Post APRV** | **-61.032^*^** | **18.085** | **11.859** | **.017** | **-111.394** | **-10.671** |  |
| Based on estimated marginal means | | | | | | | | |
| *. The mean difference is significant at the .05 level. | | | | | | | | |
| a. Dependent Variable: Time 0 PaO2. | | | | | | | | |
| c. Adjustment for multiple comparisons: Bonferroni. | | | | | | | | |

Pairwise analysis of the interaction term at each time point showed only a significant difference at Time 1 (12-24 hours after APRV).

| **Pairwise Comparisons^a^** | | | | | | | | |
| --- | --- | --- | --- | --- | --- | --- | --- | --- |
| Time | (I) Restrictive_binary | (J) Restrictive_binary | Mean Difference (I-J) | Std. Error | df | Sig.^c^ | 95% Confidence Interval for Difference^c^ | |
|  |  |  |  |  |  |  | Lower Bound | Upper Bound |
| Pre-APRV | Neuromuscular | Obesity | -12.925 | 33.742 | 9.056 | .711 | -89.184 | 63.333 |
|  | Obesity | Neuromuscular | 12.925 | 33.742 | 9.056 | .711 | -63.333 | 89.184 |
| **12-24 Post APRV** | **Neuromuscular** | **Obesity** | **-121.166^*^** | **28.202** | **9.467** | **.002** | **-184.487** | **-57.845** |
|  | **Obesity** | **Neuromuscular** | **121.166^*^** | **28.202** | **9.467** | **.002** | **57.845** | **184.487** |
| 48 hrs Post APRV | Neuromuscular | Obesity | 1.459 | 30.941 | 7.548 | .964 | -70.642 | 73.559 |
|  | Obesity | Neuromuscular | -1.459 | 30.941 | 7.548 | .964 | -73.559 | 70.642 |
| Based on estimated marginal means | | | | | | | | |
| *. The mean difference is significant at the .05 level. | | | | | | | | |
| a. Dependent Variable: Time 0 PaO2. | | | | | | | | |
| c. Adjustment for multiple comparisons: Bonferroni. | | | | | | | | |


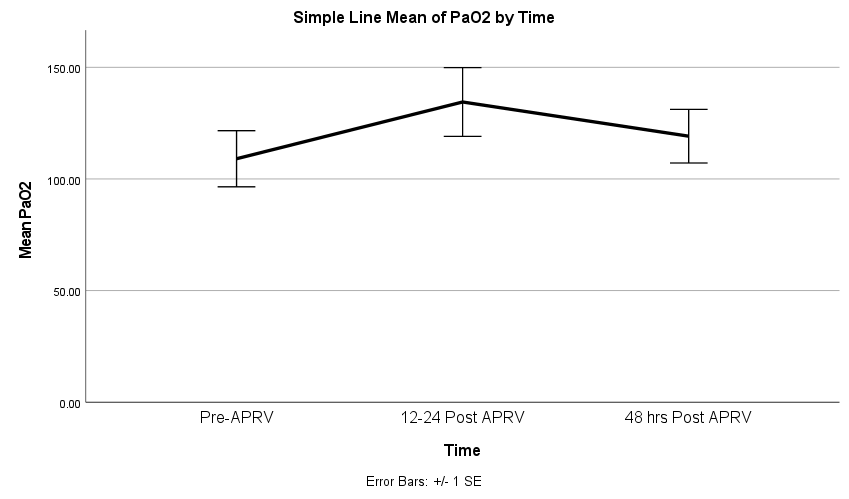


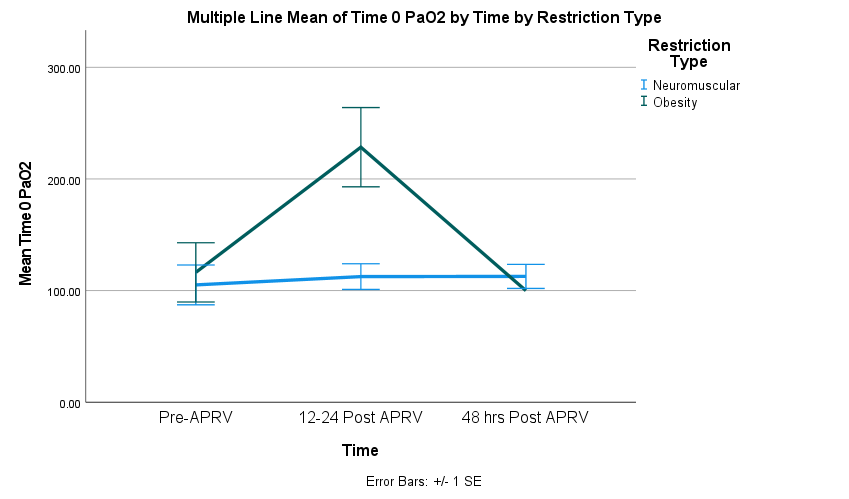


## PF Ratio

Similar to the paO2 analysis, the PF Ratio was significantly affected by Time, Group and the interaction between group and time.

| **Type III Tests of Fixed Effects^a^** | | | | |
| --- | --- | --- | --- | --- |
| Source | Numerator df | Denominator df | F | Sig. |
| Intercept | 1 | 7.715 | 296.266 | <.001 |
| **Time** | **2** | **9.973** | **6.288** | **.017** |
| **Restrictive_binary** | **1** | **7.715** | **18.502** | **.003** |
| **Restrictive_binary * Time** | **2** | **9.973** | **4.605** | **.038** |
| a. Dependent Variable: Time 0 P/F ratio. | | | | |

Pairwise analysis over time indicated that 12-24hrs Post APRV was significantly difference from Pre-APRV0 , but 48 hours post APRV and Pre-APRV were not different. Restriction type was only statistically different at the 12-24hrs Post APRV time point, but not at other time points.

| **Pairwise Comparisons^a^** | | | | | | | | |
| --- | --- | --- | --- | --- | --- | --- | --- | --- |
| (I) Time | (J) Time | Mean Difference (I-J) | Std. Error | df | Sig.^c^ | 95% Confidence Interval for Difference^c^ | |  |
|  |  |  |  |  |  | Lower Bound | Upper Bound |  |
| Pre-APRV | **12-24 Post APRV** | **-193.339^*^** | **55.996** | **6.858** | **.033** | **-369.540** | **-17.137** |  |
|  | 48 hrs Post APRV | -40.939 | 41.947 | 11.286 | 1.000 | -158.709 | 76.831 |  |
| 12-24 Post APRV | **Pre-APRV** | **193.339^*^** | **55.996** | **6.858** | **.033** | **17.137** | **369.540** |  |
|  | 48 hrs Post APRV | 152.400 | 51.048 | 7.017 | .061 | -7.142 | 311.942 |  |
| 48 hrs Post APRV | Pre-APRV | 40.939 | 41.947 | 11.286 | 1.000 | -76.831 | 158.709 |  |
|  | 12-24 Post APRV | -152.400 | 51.048 | 7.017 | .061 | -311.942 | 7.142 |  |
| Based on estimated marginal means | | | | | | | | |
| *. The mean difference is significant at the .05 level. | | | | | | | | |
| a. Dependent Variable: Time 0 P/F ratio. | | | | | | | | |
| c. Adjustment for multiple comparisons: Bonferroni. | | | | | | | | |

| **Pairwise Comparisons^a^** | | | | | | | | |
| --- | --- | --- | --- | --- | --- | --- | --- | --- |
| Time | (I) Restrictive_binary | (J) Restrictive_binary | Mean Difference (I-J) | Std. Error | df | Sig.^c^ | 95% Confidence Interval for Difference^c^ | |
|  |  |  |  |  |  |  | Lower Bound | Upper Bound |
| Pre-APRV | Neuromuscular | Obesity | -64.984 | 66.757 | 7.643 | .360 | -220.189 | 90.221 |
|  | Obesity | Neuromuscular | 64.984 | 66.757 | 7.643 | .360 | -90.221 | 220.189 |
| **12-24 Post APRV** | **Neuromuscular** | **Obesity** | **-323.061^*^** | **73.445** | **5.775** | **.005** | **-504.483** | **-141.640** |
|  | **Obesity** | **Neuromuscular** | **323.061^*^** | **73.445** | **5.775** | **.005** | **141.640** | **504.483** |
| 48 hrs Post APRV | Neuromuscular | Obesity | -14.533 | 55.411 | 4.348 | .805 | -163.643 | 134.578 |
|  | Obesity | Neuromuscular | 14.533 | 55.411 | 4.348 | .805 | -134.578 | 163.643 |
| Based on estimated marginal means | | | | | | | | |
| *. The mean difference is significant at the .05 level. | | | | | | | | |
| a. Dependent Variable: Time 0 P/F ratio. | | | | | | | | |
| c. Adjustment for multiple comparisons: Bonferroni. | | | | | | | | |


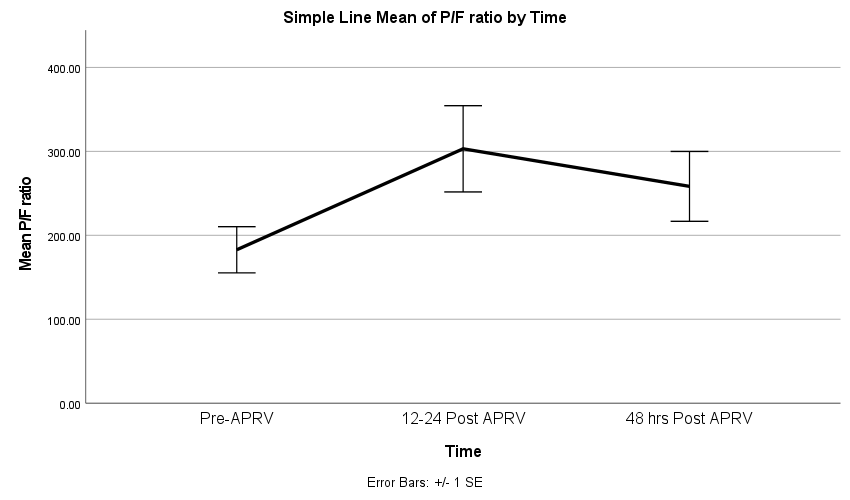


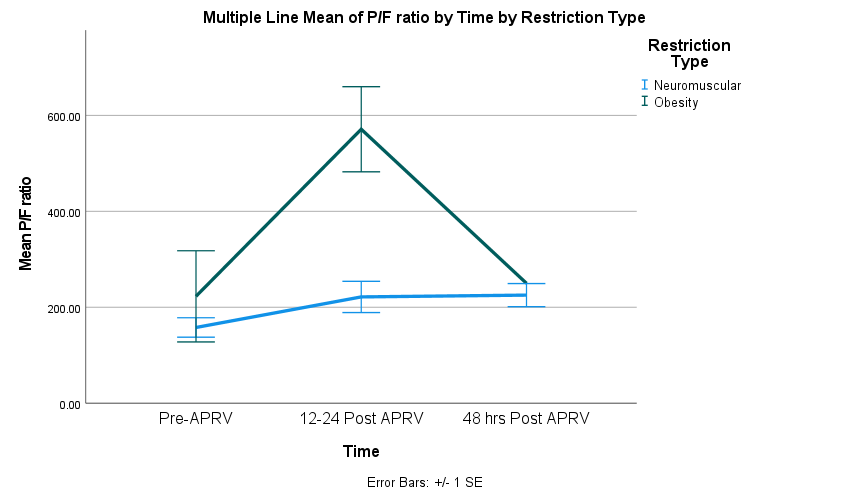


## Oxygenation Index

For the OI, there was no impact on the model from Restriction Type, Time or the interaction between restriction type and time..

| **Type III Tests of Fixed Effects^a^** | | | | |
| --- | --- | --- | --- | --- |
| Source | Numerator df | Denominator df | F | Sig. |
| Intercept | 1 | 7.798 | 33.696 | <.001 |
| Time | 2 | 8.624 | 4.263 | .052 |
| Restrictive_binary | 1 | 7.798 | .198 | .668 |
| Restrictive_binary * Time | 2 | 8.624 | 1.925 | .204 |
| a. Dependent Variable: Time 0 OI. | | | | |

There were no pairwise differences over time or between groups.

| **Pairwise Comparisons^a^** | | | | | | | | |
| --- | --- | --- | --- | --- | --- | --- | --- | --- |
| (I) Time | (J) Time | Mean Difference (I-J) | Std. Error | df | Sig.^b^ | 95% Confidence Interval for Difference^b^ | |  |
|  |  |  |  |  |  | Lower Bound | Upper Bound |  |
| Pre-APRV | 12-24 Post APRV | 9.839 | 3.439 | 7.445 | .068 | -.729 | 20.407 |  |
|  | 48 hrs Post APRV | 8.742 | 3.792 | 8.629 | .143 | -2.488 | 19.971 |  |
| 12-24 Post APRV | Pre-APRV | -9.839 | 3.439 | 7.445 | .068 | -20.407 | .729 |  |
|  | 48 hrs Post APRV | -1.098 | 1.685 | 8.681 | 1.000 | -6.080 | 3.885 |  |
| 48 hrs Post APRV | Pre-APRV | -8.742 | 3.792 | 8.629 | .143 | -19.971 | 2.488 |  |
|  | 12-24 Post APRV | 1.098 | 1.685 | 8.681 | 1.000 | -3.885 | 6.080 |  |
| Based on estimated marginal means | | | | | | | | |
| a. Dependent Variable: Time 0 OI. | | | | | | | | |
| b. Adjustment for multiple comparisons: Bonferroni. | | | | | | | | |

| **Pairwise Comparisons^a^** | | | | | | | | |
| --- | --- | --- | --- | --- | --- | --- | --- | --- |
| Time | (I) Restrictive_binary | (J) Restrictive_binary | Mean Difference (I-J) | Std. Error | df | Sig.^b^ | 95% Confidence Interval for Difference^b^ | |
|  |  |  |  |  |  |  | Lower Bound | Upper Bound |
| Pre-APRV | Neuromuscular | Obesity | -3.414 | 7.464 | 6.950 | .661 | -21.089 | 14.261 |
|  | Obesity | Neuromuscular | 3.414 | 7.464 | 6.950 | .661 | -14.261 | 21.089 |
| 12-24 Post APRV | Neuromuscular | Obesity | 6.304 | 3.160 | 6.337 | .091 | -1.329 | 13.936 |
|  | Obesity | Neuromuscular | -6.304 | 3.160 | 6.337 | .091 | -13.936 | 1.329 |
| 48 hrs Post APRV | Neuromuscular | Obesity | 1.602 | 2.638 | 4.722 | .572 | -5.301 | 8.505 |
|  | Obesity | Neuromuscular | -1.602 | 2.638 | 4.722 | .572 | -8.505 | 5.301 |
| Based on estimated marginal means | | | | | | | | |
| a. Dependent Variable: Time 0 OI. | | | | | | | | |
| b. Adjustment for multiple comparisons: Bonferroni. | | | | | | | | |


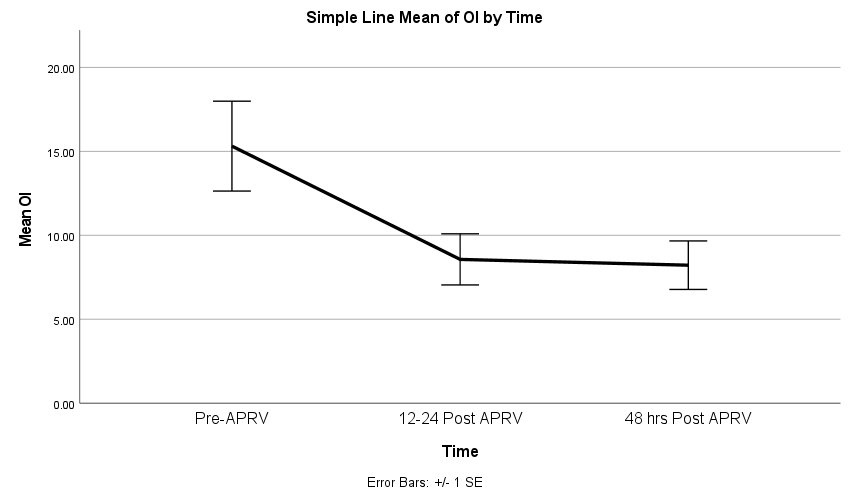


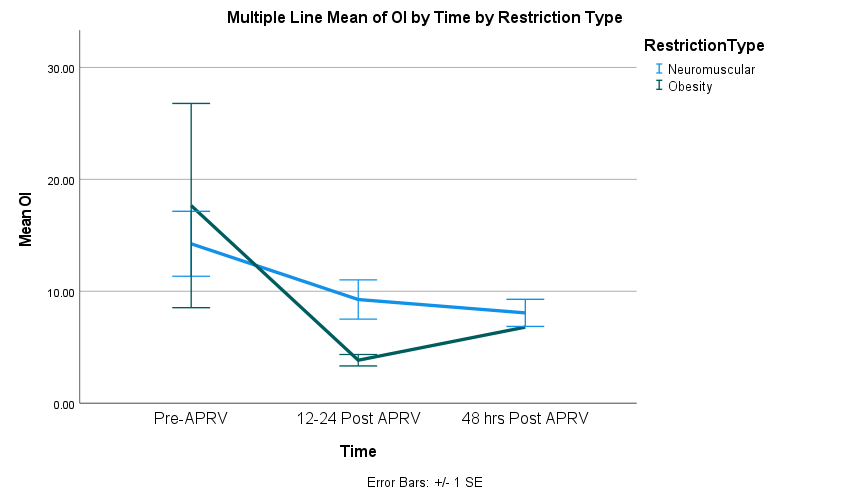


## pCO_2_

There were no effects for pCO2.

| **Type III Tests of Fixed Effects^a^** | | | | |
| --- | --- | --- | --- | --- |
| Source | Numerator df | Denominator df | F | Sig. |
| Intercept | 1 | 14.643 | 828.685 | <.001 |
| Time | 2 | 18.896 | 2.188 | .140 |
| Restrictive_binary | 1 | 14.643 | 2.204 | .159 |
| Restrictive_binary * Time | 2 | 18.896 | .127 | .882 |
| a. Dependent Variable: Time 0 PCO2. | | | | |

There was no difference in the time points or between groups at each time point.

| **Pairwise Comparisons^a^** | | | | | | | | |
| --- | --- | --- | --- | --- | --- | --- | --- | --- |
| (I) Time | (J) Time | Mean Difference (I-J) | Std. Error | df | Sig.^b^ | 95% Confidence Interval for Difference^b^ | |  |
|  |  |  |  |  |  | Lower Bound | Upper Bound |  |
| Pre-APRV | 12-24 Post APRV | -3.011 | 2.737 | 17.616 | .858 | -10.250 | 4.227 |  |
|  | 48 hrs Post APRV | -6.767 | 3.244 | 24.016 | .143 | -15.115 | 1.580 |  |
| 12-24 Post APRV | Pre-APRV | 3.011 | 2.737 | 17.616 | .858 | -4.227 | 10.250 |  |
|  | 48 hrs Post APRV | -3.756 | 2.759 | 19.872 | .566 | -10.967 | 3.455 |  |
| 48 hrs Post APRV | Pre-APRV | 6.767 | 3.244 | 24.016 | .143 | -1.580 | 15.115 |  |
|  | 12-24 Post APRV | 3.756 | 2.759 | 19.872 | .566 | -3.455 | 10.967 |  |
| Based on estimated marginal means | | | | | | | | |
| a. Dependent Variable: Time 0 PCO2. | | | | | | | | |
| b. Adjustment for multiple comparisons: Bonferroni. | | | | | | | | |

| **Pairwise Comparisons^a^** | | | | | | | | |
| --- | --- | --- | --- | --- | --- | --- | --- | --- |
| Time | (I) Restrictive_binary | (J) Restrictive_binary | Mean Difference (I-J) | Std. Error | df | Sig.^b^ | 95% Confidence Interval for Difference^b^ | |
|  |  |  |  |  |  |  | Lower Bound | Upper Bound |
| Pre-APRV | Neuromuscular | Obesity | -6.818 | 4.942 | 14.048 | .189 | -17.415 | 3.778 |
|  | Obesity | Neuromuscular | 6.818 | 4.942 | 14.048 | .189 | -3.778 | 17.415 |
| 12-24 Post APRV | Neuromuscular | Obesity | -4.295 | 4.636 | 13.001 | .371 | -14.310 | 5.719 |
|  | Obesity | Neuromuscular | 4.295 | 4.636 | 13.001 | .371 | -5.719 | 14.310 |
| 48 hrs Post APRV | Neuromuscular | Obesity | -4.004 | 4.775 | 11.023 | .420 | -14.512 | 6.504 |
|  | Obesity | Neuromuscular | 4.004 | 4.775 | 11.023 | .420 | -6.504 | 14.512 |
| Based on estimated marginal means | | | | | | | | |
| a. Dependent Variable: Time 0 PCO2. | | | | | | | | |
| b. Adjustment for multiple comparisons: Bonferroni. | | | | | | | | |


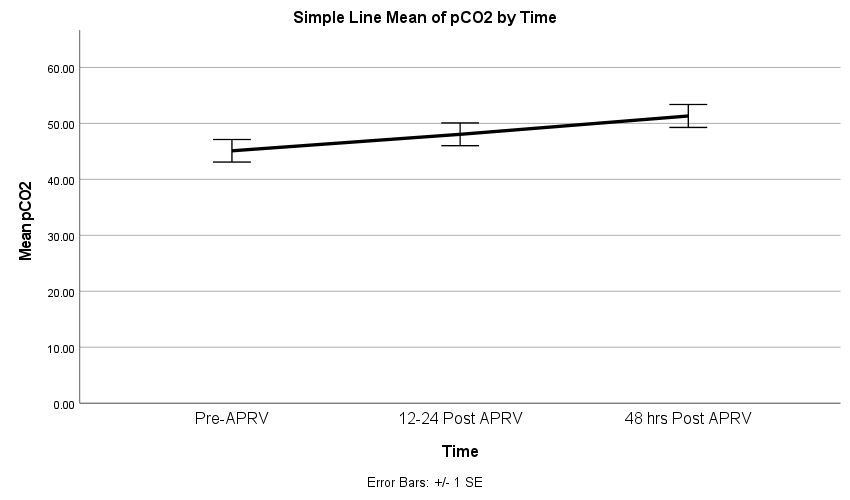


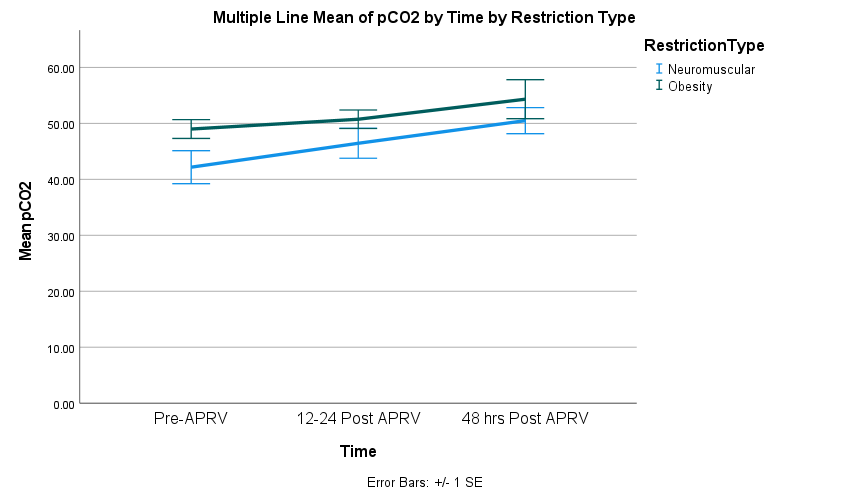


## Percent Atelectisis

For percent atelectasis, there was a significant effect of time.

| **Type III Tests of Fixed Effects^a^** | | | | |
| --- | --- | --- | --- | --- |
| Source | Numerator df | Denominator df | F | Sig. |
| Intercept | 1 | 18.425 | 85.245 | <.001 |
| **Time** | **2** | **13.271** | **8.137** | **.005** |
| Restrictive_binary | 1 | 18.425 | 1.388 | .254 |
| Restrictive_binary * Time | 2 | 13.271 | 3.118 | .078 |
| a. Dependent Variable: Time 0 Percent Atelectisis. | | | | |

There was a significant difference between Pre-APRV an the 12-24 and 48hrs post APRV times, but not between 12-24 and 48 hrs post APRV. There was a significant difference between Restriction Types at 12-24 Post APRV.

| **Pairwise Comparisons^a^** | | | | | | | | |
| --- | --- | --- | --- | --- | --- | --- | --- | --- |
| (I) Time | (J) Time | Mean Difference (I-J) | Std. Error | df | Sig.^c^ | 95% Confidence Interval for Difference^c^ | |  |
|  |  |  |  |  |  | Lower Bound | Upper Bound |  |
| **Pre-APRV** | **12-24 Post APRV** | **.145^*^** | **.038** | **16.895** | **.005** | **.043** | **.247** |  |
|  | **48 hrs Post APRV** | **.142^*^** | **.041** | **18.318** | **.009** | **.033** | **.251** |  |
| 12-24 Post APRV | **Pre-APRV** | **-.145^*^** | **.038** | **16.895** | **.005** | **-.247** | **-.043** |  |
|  | 48 hrs Post APRV | -.003 | .035 | 7.946 | 1.000 | -.110 | .103 |  |
| 48 hrs Post APRV | **Pre-APRV** | **-.142^*^** | **.041** | **18.318** | **.009** | **-.251** | **-.033** |  |
|  | 12-24 Post APRV | .003 | .035 | 7.946 | 1.000 | -.103 | .110 |  |
| Based on estimated marginal means | | | | | | | | |
| *. The mean difference is significant at the .05 level. | | | | | | | | |
| a. Dependent Variable: Time 0 Percent Atelectasis. | | | | | | | | |
| c. Adjustment for multiple comparisons: Bonferroni. | | | | | | | | |

| **Pairwise Comparisons^a^** | | | | | | | | |
| --- | --- | --- | --- | --- | --- | --- | --- | --- |
| Time | (I) Restrictive_binary | (J) Restrictive_binary | Mean Difference (I-J) | Std. Error | df | Sig.^b^ | 95% Confidence Interval for Difference^b^ | |
|  |  |  |  |  |  |  | Lower Bound | Upper Bound |
| Pre-APRV | Neuromuscular | Obesity | -.077 | .062 | 12.312 | .239 | -.211 | .058 |
|  | Obesity | Neuromuscular | .077 | .062 | 12.312 | .239 | -.058 | .211 |
| **12-24 Post APRV** | **Neuromuscular** | **Obesity** | **.047** | **.036** | **12.119** | **.217** | **-.031** | **.124** |
|  | **Obesity** | **Neuromuscular** | **-.047** | **.036** | **12.119** | **.217** | **-.124** | **.031** |
| 48 hrs Post APRV | Neuromuscular | Obesity | .130 | .056 | 5.693 | .063 | -.010 | .270 |
|  | Obesity | Neuromuscular | -.130 | .056 | 5.693 | .063 | -.270 | .010 |
| Based on estimated marginal means | | | | | | | | |
| a. Dependent Variable: Time 0 Percent Atelectasis. | | | | | | | | |
| b. Adjustment for multiple comparisons: Bonferroni. | | | | | | | | |


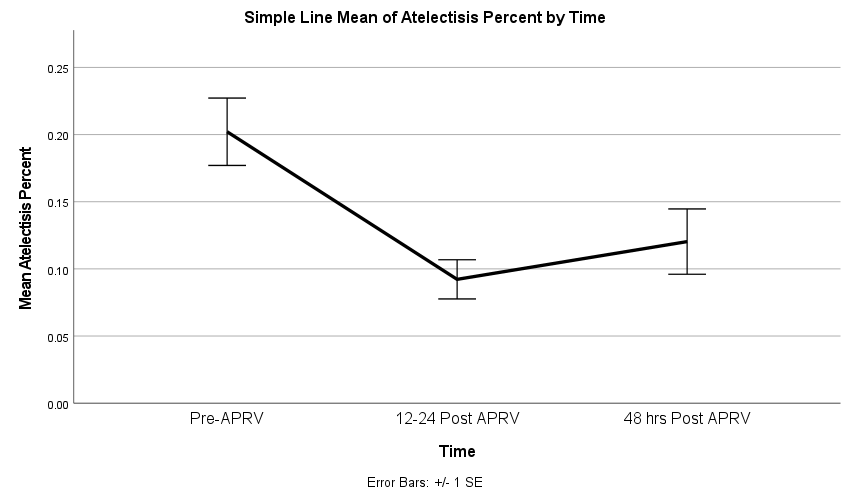


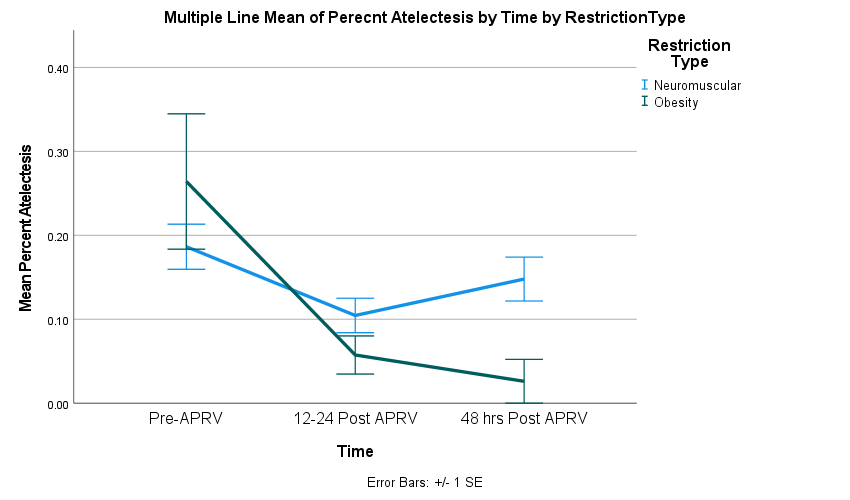


## Lung Area

For lung area, there were significant effects of time, and the interaction between time and restriction type.

| **Type III Tests of Fixed Effects^a^** | | | | |
| --- | --- | --- | --- | --- |
| Source | Numerator df | Denominator df | F | Sig. |
| Intercept | 1 | 13.340 | 123.538 | <.001 |
| **Time** | **2** | **12.617** | **16.867** | **<.001** |
| Restrictive_binary | 1 | 13.340 | 2.094 | .171 |
| **Restrictive_binary * Time** | **2** | **12.617** | **14.771** | **<.001** |
| a. Dependent Variable: Total area of the lungs1. | | | | |

For pairwise comparisons, only the Pre-APRV and 12-24 Post APRV was significantly different as was the Restriction type at the 12-24 hr Post APRV time point.

| **Pairwise Comparisons^a^** | | | | | | | |
| --- | --- | --- | --- | --- | --- | --- | --- |
| (I) Time | (J) Time | Mean Difference (I-J) | Std. Error | df | Sig.^c^ | 95% Confidence Interval for Difference^c^ | |
|  |  |  |  |  |  | Lower Bound | Upper Bound |
| Pre-APRV | **12-24 Post APRV** | **-7644.367^*^** | **1365.390** | **17.784** | **<.001** | **-11252.289** | **-4036.446** |
|  | 48 hrs Post APRV | -4232.926 | 2546.581 | 12.092 | .366 | -11302.557 | 2836.705 |
| **12-24 Post APRV** | **Pre-APRV** | **7644.367^*^** | **1365.390** | **17.784** | **<.001** | **4036.446** | **11252.289** |
|  | 48 hrs Post APRV | 3411.441 | 2158.127 | 10.964 | .427 | -2678.061 | 9500.942 |
| 48 hrs Post APRV | Pre-APRV | 4232.926 | 2546.581 | 12.092 | .366 | -2836.705 | 11302.557 |
|  | 12-24 Post APRV | -3411.441 | 2158.127 | 10.964 | .427 | -9500.942 | 2678.061 |
| Based on estimated marginal means | | | | | | | |
| *. The mean difference is significant at the .05 level. | | | | | | | |
| a. Dependent Variable: Total area of the lungs1. | | | | | | | |
| c. Adjustment for multiple comparisons: Bonferroni. | | | | | | | |

| **Pairwise Comparisons^a^** | | | | | | | | |
| --- | --- | --- | --- | --- | --- | --- | --- | --- |
| Time | (I) Restrictive_binary | (J) Restrictive_binary | Mean Difference (I-J) | Std. Error | df | Sig.^c^ | 95% Confidence Interval for Difference^c^ | |
|  |  |  |  |  |  |  | Lower Bound | Upper Bound |
| Pre-APRV | Neuromuscular | Obesity | -975.101 | 4881.223 | 13.024 | .845 | -11518.386 | 9568.184 |
|  | Obesity | Neuromuscular | 975.101 | 4881.223 | 13.024 | .845 | -9568.184 | 11518.386 |
| **12-24 Post APRV** | **Neuromuscular** | **Obesity** | **-15023.367^*^** | **5510.042** | **12.598** | **.018** | **-26965.796** | **-3080.937** |
|  | **Obesity** | **Neuromuscular** | **15023.367^*^** | **5510.042** | **12.598** | **.018** | **3080.937** | **26965.796** |
| 48 hrs Post APRV | Neuromuscular | Obesity | -7314.573 | 7047.413 | 12.283 | .319 | -22630.429 | 8001.282 |
|  | Obesity | Neuromuscular | 7314.573 | 7047.413 | 12.283 | .319 | -8001.282 | 22630.429 |
| Based on estimated marginal means | | | | | | | | |
| *. The mean difference is significant at the .05 level. | | | | | | | | |
| a. Dependent Variable: Total area of the lungs1. | | | | | | | | |
| c. Adjustment for multiple comparisons: Bonferroni. | | | | | | | | |


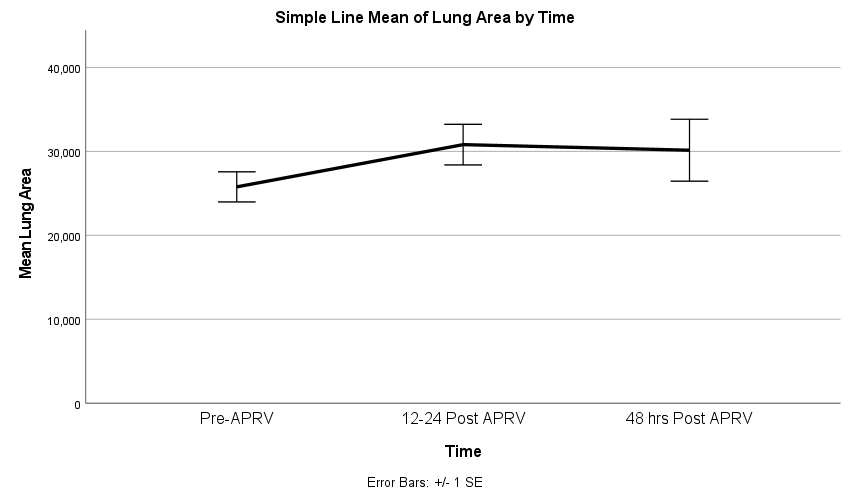


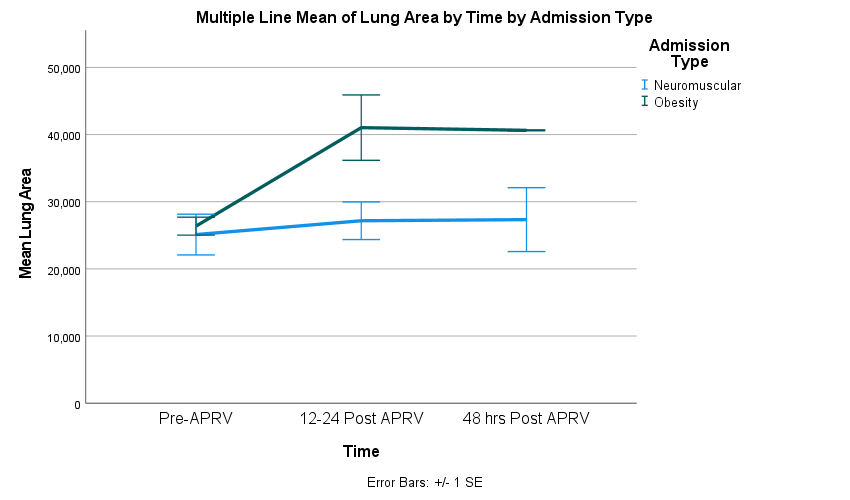

Supplement: Supplementary file 2 [file Datasheet1.docx]
